# Distribution of Suicin Gene Clusters in* Streptococcus suis* Serotype 2 Belonging to Sequence Types 25 and 28

**DOI:** 10.1155/2016/6815894

**Published:** 2016-12-18

**Authors:** Taryn B. T. Athey, Katy Vaillancourt, Michel Frenette, Nahuel Fittipaldi, Marcelo Gottschalk, Daniel Grenier

**Affiliations:** ^1^Public Health Ontario Laboratory, Toronto, ON, Canada; ^2^Groupe de Recherche en Écologie Buccale (GREB), Faculté de Médecine Dentaire, Université Laval, Quebec City, QC, Canada; ^3^Centre de Recherche en Infectiologie Porcine et Avicole (CRIPA), Fonds de Recherche du Québec-Nature et Technologies (FQRNT), Saint-Hyacinthe, QC, Canada; ^4^Department of Laboratory Medicine and Pathobiology, Faculty of Medicine, University of Toronto, Toronto, ON, Canada; ^5^Groupe de Recherche sur les Maladies Infectieuses du Porc (GREMIP), Faculté de Médecine Vétérinaire, Université de Montréal, Saint-Hyacinthe, QC, Canada

## Abstract

Recently, we reported the purification and characterization of three distinct lantibiotics (named suicin 90-1330, suicin 3908, and suicin 65) produced by* Streptococcus suis*. In this study, we investigated the distribution of the three suicin lantibiotic gene clusters among serotype 2* S. suis* strains belonging to sequence type (ST) 25 and ST28, the two dominant STs identified in North America. The genomes of 102 strains were interrogated for the presence of suicin gene clusters encoding suicins 90-1330, 3908, and 65. The gene cluster encoding suicin 65 was the most prevalent and mainly found among ST25 strains. In contrast, none of the genes related to suicin 90-1330 production were identified in 51 ST25 strains nor in 35/51 ST28 strains. However, the complete suicin 90-1330 gene cluster was found in ten ST28 strains, although some genes in the cluster were truncated in three of these isolates. The vast majority (101/102) of* S. suis *strains did not possess any of the genes encoding suicin 3908. In conclusion, this study indicates heterogeneous distribution of suicin genes in* S. suis*.

## 1. Introduction

Bacteriocins are cationic bactericidal peptides that primarily act by disrupting the cell membrane integrity of the target bacteria [1]. More specifically, lantibiotics are an important family of heat stable low molecular weight bacteriocins that possess unusual posttranslationally modified amino acids, such as lanthionine, methyllanthionine, dehydroalanine, and dehydrobutyrine with thioether linkages [2]. Nisin A, produced by* Lactococcus lactis *subsp.* lactis* and so far the most studied lantibiotic, was approved as GRAS (Generally Recognized As Safe) by the US Food and Drug Administration (FDA) in 1988 and is currently used commercially in more than 50 countries as a food preservative, especially for dairy products [3]. The lantibiotic biosynthesis machinery involves many enzymes which are encoded by genes organized in gene clusters. Typically, lantibiotic gene clusters include a structural gene encoding a prelantibiotic peptide, as well as genes required for the modification of amino acids, maturation, export, regulation, and immunity [2]. More specifically, the structural gene encodes a prepeptide containing a leader sequence at the N-terminus, which is ultimately cleaved, and a propeptide at the C-terminus in which many or all of the serine and threonine residues are modified [2].

*Streptococcus suis* is a major swine pathogen responsible for a wide array of infections including life-threatening meningitis, septicemia, and pneumonia [4]. In addition, the organism is an emerging agent of zoonotic disease, particularly in some Asian countries where the general population is at risk [5]. Of the 35 serotypes (1 to 34 and 1/2) that have been originally described based on the composition and structure of the capsular polysaccharides, serotype 2 is the most frequently associated with disease [4, 6]. In the last decade, other serotypes with particular geographical distributions have also been identified as the source of many infections [6]. For instance, in North America, serotypes 2 and 3 show a prevalence of 24.3% and 21%, respectively, followed by serotypes 1/2, 8, and 7 [6].* S. suis* serotype 2 can be genotyped using multilocus sequence typing (MLST) [7]. In North America, three genotypes have been described among* S. suis *serotype 2 strains; the relatively frequent sequence type (ST) 25 and ST28 and the less frequent ST1 [8]. Animal infection models have shown that strains belonging to ST1 are highly virulent whereas ST25 and ST28 isolates have an intermediate and low virulence, respectively [8].

Recently, we purified and characterized three different lantibiotics, named suicins, produced by three distinct strains of* S. suis *serotype 2 [9–11]. The mature peptides corresponding to each suicin showed poor identity (≤24%) between each other, thus indicating that they are unrelated. Suicin 90-1330 (SslA) is a type A (linear) lantibiotic secreted by a nonvirulent (in both mouse and pig infection models) isolate of* S. suis *(strain 90-1330) belonging to ST28 and exhibits high homology (90.9%) with nisin U produced by* Streptococcus uberis* [9]. The production of suicin 90-1330 involves a gene cluster comprising eleven genes [9]. Suicin 3908 (SuiA) is a type B (N-terminal linear and C-terminal globular moieties) lantibiotic produced by a nonvirulent (newborn germ-free pig infection model) isolate of* S. suis *(strain 3908; unknown ST) isolated from a healthy carrier pig, and shows some identity with bovicin HJ50 (*Streptococcus bovis*; 49.2% identity) [10]. The suicin 3908 gene cluster contains nine genes [10]. Lastly, a second type B lantibiotic (suicin 65; SssA) showing a high identity with lantibiotics produced by* Streptococcus pyogenes *(streptococcin FF22, 84.6% identity) and* L. lactis *subsp.* lactis* (lacticin 481, 74.1% identity) was identified and characterized in* S. suis* 65, an ST28 avirulent (newborn germ-free pig infection model) strain isolated from a healthy carrier pig [11]. The gene cluster involved in the production of suicin 65 was found to contain ten genes, including a duplication of the structural gene. Since all three suicins were found to be bactericidal for highly virulent ST1 strains of* S. suis *[9–11], the use of the purified lantibiotics or the lantibiotic-producing strains may represent a valuable strategy to control* S. suis *infections and for reducing antibiotic use in the swine industry and consequently the spread of antibiotic resistance. However, the prevalence of the different suicin gene clusters among the circulating population of* S. suis *strains is not known. In this study, we investigated the distribution and genetic diversity of suicin gene clusters in* S. suis* serotype 2 belonging to ST25 and ST28, the two dominant STs found in North America.

## 2. Materials and Methods

### 2.1. Bacterial Strains

One hundred and two strains of* S. suis *serotype 2 belonging to either ST25 (*n* = 51) or ST28 (*n* = 51) were included in this study (Table 1). These strains were isolated from diseased pigs in Canada, the United States, Japan, and Thailand and have been described previously [12, 13].

### 2.2. Genome Analysis

**Figure 1:**
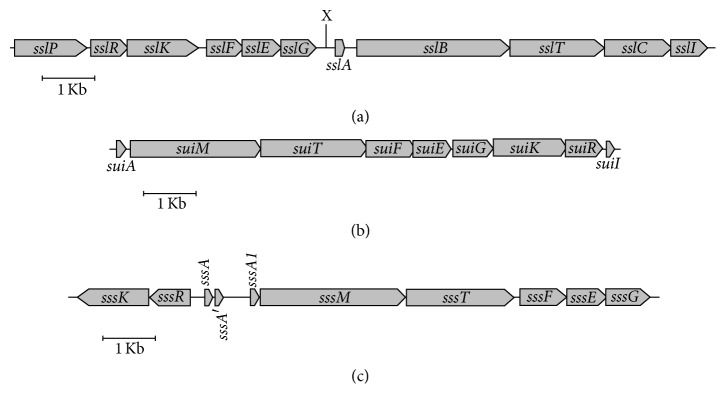
Genetic organization of the suicin 90-1330 (a), 3908 (b), and 65 (c) gene clusters.* sslA/suiA/sssA/sssA*′*/sssA1*: suicin precursor;* sslB*: dehydratase involved in suicin synthesis;* sslC*: cyclase involved in suicin synthesis;* sslE/suiE/sssE*,* sslF/suiF/sssF*,* sslG/suiG/sssG*, and* sslI/suiI*: immunity proteins;* sslK/suiK/sssK*: sensor histidine kinase;* suiM/sssM*: synthetase involved in lantibiotic modification;* sslP*: protease involved in proteolytic cleavage of the leader peptide;* sslR/suiR/sssR*: response regulator;* sslT/suiT/sssT*: ABC transporter.

The genomes of the 102 strains were sequenced as paired-end reads with either a HiSeq 2500 or a MiSeq instrument (Illumina, San Diego, CA, USA) as described in previous reports [12, 13]. Sequences with accession numbers SRP065686 and SRP058193, for ST25 and ST28 strains, respectively, were retrieved from the NCBI sequence read archive (SRA). The A5 pipeline was used for de novo assembly of Illumina sequenced strains [14]. Contigs were ordered relative to the reference genome NSUI060 (accession number: CP012911) or NSUI002 (accession number: CP011419), for ST25 and ST28, respectively, using Progressive Mauve [15]. Pseudochromosomes were next created by concatenating the ordered contigs using the sequence NNNNNCATTCCATTCATTAATTAATTAATGAATGAATGNNNNN, which introduces start and stop codons in all 6 reading frames, as a separator. Pseudochromosomes and the genome sequences of reference strains NSUI002 and NSUI060 were converted into queryable BLASTN databases using BLAST+. Gene clusters encoding suicins 90-1330 (accession number: KU867866), 3908 (accession number: KU867867), and 65 (accession number: KU867868) have been described previously and are illustrated in Figure 1 [9–11]. The sequences of individual genes, as well as the full regions for each suicin cluster, were blasted against each genome and pseudochromosome database to test for the presence of genes encoding suicins 90-1330, 3908, and 65. The presence of putative promoter sequences upstream the genes coding for immunity proteins was analyzed with the bacterial promoter recognition program BProm (http://linux1.softberry.com) [16].

### 2.3. Plate Diffusion Assay for Detecting Suicin Susceptibility

Overnight cultures of the suicin-producing* S. suis* strains (90-1330, 3908, and 65) were spotted (2 *μ*L) on Todd-Hewitt broth (THB; BD-Canada, Mississauga, ON, Canada) agar plates. After a 24 h incubation at 37°C to allow growth, the plates were overlaid with soft THB agar (0.75% agar, w/v) that had been inoculated (700 *μ*L of culture/7 mL of agar) with a 24 h culture of* S. suis *NSUI018 (possessing immunity gene for suicin 90-1330), NSUI036 (possessing immunity gene for suicin 3908), and NSUI060 (possessing immunity gene for suicin 65). The plates were further incubated for 24 h at 37°C, and the presence of an inhibitory zone was observed.

## 3. Results and Discussion

**Figure 2:**
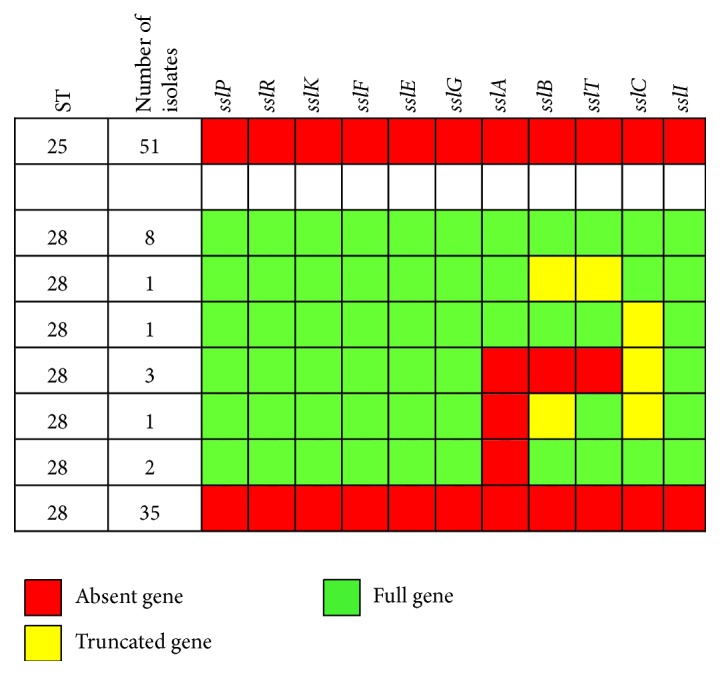
Distribution of suicin 90-1330 gene cluster among* S. suis* serotype 2 strains of ST25 and ST28.* sslA*: suicin precursor;* sslB*: dehydratase involved in suicin synthesis;* sslC*: cyclase involved in suicin synthesis;* sslE*,* sslF*,* sslG*, and* sslI*: immunity proteins;* sslK*: sensor histidine kinase;* sslP*: protease involved in proteolytic cleavage of the leader peptide;* sslR*: response regulator;* sslT*: ABC transporter.

Suicin 90-1330 was originally identified in* S. suis* strain 90-1330, which belongs to ST28 [9]. Interestingly, 35 out of 51 ST28 strains analyzed did not possess any of the genes involved in the production of this suicin (Figure 2). On the other hand, all genes expected in a complete suicin 90-1330 gene cluster were identified in ten ST28 strains, while the locus was partially present in six ST28 strains. However, it is worth mentioning that a full, uninterrupted suicin 90-1330 gene cluster was identified in only seven of these ten ST28 strains (i.e., all genes were identified as a continuum of genes similar to that of Figure 1 in a single contig of the de novo assembled genomes). On the other hand, in one of the ST28 strains that had all suicin 90-1330 genes, the genes were spread across 3 contigs, suggesting two transposon insertions in intergenic regions of the suicin 90-1330 gene cluster. In two other ST28 strains, genes were spread across two contigs, apparently by insertion of a transposon that truncated parts of gene* sslT* (one strain) or gene* sslC* (one strain). These transposon insertions should result in strains that are unable to express the suicin. In the six strains in which seven or more suicin 90-1330 cluster genes were only partially present (Figure 2), the gene specifically encoding the structural suicin (*sslA*) was missing, suggesting that this suicin cannot be produced by these strains. In addition, in 50% of strains with a partial gene cluster, the genes were spread across two contigs (Table 1), probably due to transposon insertions. None of the ST25 strains analyzed in this study possessed genes encoding suicin 90-1330 (Figure 2).

**Figure 3:**
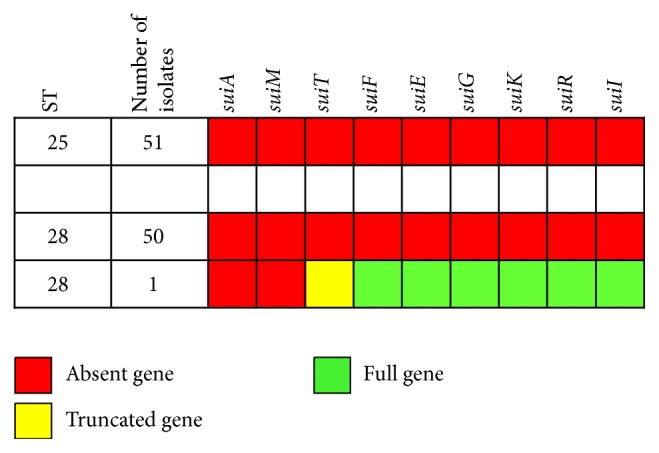
Distribution of suicin 3908 gene cluster among* S. suis* serotype 2 strains of ST25 and ST28.* suiA*: suicin precursor;* suiE*,* suiF*,* suiG*, and* suiI*: immunity proteins;* suiK*: sensor histidine kinase;* suiM*: synthetase involved in lantibiotic modification;* suiR*: response regulator;* suiT*: ABC transporter.

Examination of the genome data revealed that none of the ST25 strains possessed the suicin 3908 gene cluster. Similar results were also observed in 50 of the 51 ST28 isolates (Figure 3). One ST28 strain (NSUI036, isolated in Thailand) possessed some of the cluster genes (in a single contig, Table 1) but lacked the first two genes,* suiA* (structural gene) and* suiM*, in full and* suiT* in part.

**Figure 4:**
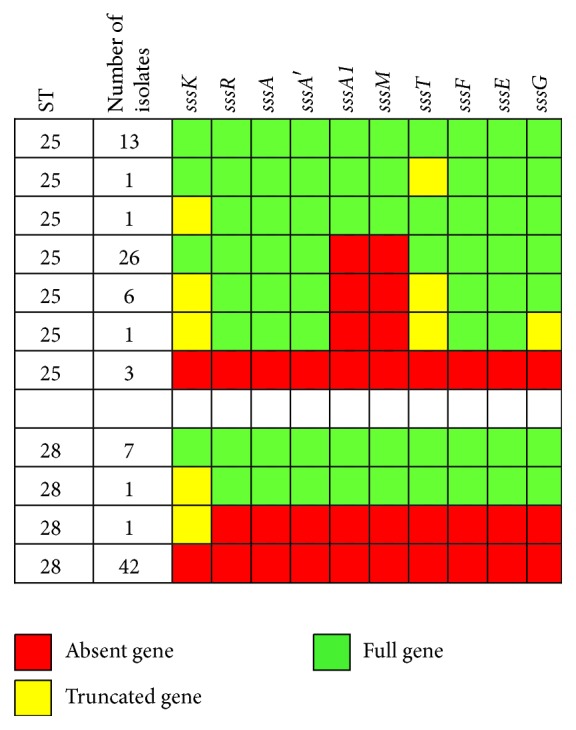
Distribution of suicin 65 gene cluster among* S. suis* serotype 2 strains of ST25 and ST28.* sssA/sssA*′*/sssA1*: suicin precursor;* sssE*,* sssF*, and* sssG*: immunity proteins;* sssK*: sensor histidine kinase;* sssM*: synthetase involved in lantibiotic modification;* sssR*: response regulator;* sssT*: ABC transporter.

The suicin 65 gene cluster was found in its entirety in 15 of the 51 ST25 strains and partially in 33 of the 51 ST25 strains (Figure 4). Only three ST25 strains did not possess any of the genes of the suicin 65 gene cluster. In the 33 ST25 strains that possessed a partial suicin 65 gene cluster, the structural* sssA1* gene and gene* sssM *were always absent, thus suggesting that these strains cannot produce active suicin 65. The majority of ST28 strains (42/51) did not possess any of the suicin 65 cluster genes. However, eight of the 51 strains possessed all cluster genes. One ST28 strain had only the* sssK *gene, which lies at the extremity of the cluster. Of the ST25 and ST28 strains that contained the partial or full suicin 65 cluster, 81% (ST25) and 88% (ST28) contained all genes in a single contig (Table 1). In two of the ST25 and ST28 strains that had all suicin 65 genes, the genes were spread across 2 contigs (Table 1).

The in silico analysis described above identified strains which possessed a partial suicin locus containing genes encoding the immunity proteins but no gene encoding the structural suicin protein. More specifically, strain NSUI018 possessed immunity protein for suicin 90-1330, strain NSUI036 possessed immunity protein for suicin 3908, and strain NSUI060 possessed immunity protein for suicin 65. The genomes of these three strains were further analyzed for the presence of promoter-like regions upstream of the genes encoding the immunity proteins. This additional analysis revealed the presence of putative promoter sequences upstream of the gene encoding the immunity proteins in all three strains. However, as demonstrated by a plate diffusion assay, all three strains were found to be susceptible to the strain producing the corresponding suicin (data not shown). Incorporation of CaCO_3_ ruled out the possibility that inhibition could be related to acid production. Thus, putative sequences identified in silico upstream of the immunity genes are not functional promoters or additional factors are required to allow immunity to suicins. With the perspective to use suicin-producing avirulent strains in prophylaxis, such results indicate that the presence of a partial suicin locus containing the immunity genes does not endow a strain with suicin resistance.

## 4. Conclusions

The gene clusters encoding suicin 65 (mostly in ST25 strains) and, to a lesser extent, suicin 90-1330 (exclusively in ST28 strains) were the most prevalent. None of the strains that had suicin 90-1330 had suicin 65 and vice versa.

## Figures and Tables

**Table 1:** Presence and absence of suicin regions and genes among *S. suis* serotype 2 strains of ST25 and ST28.

Strain	Sequence type	Suicin 65		Suicin 90-1330		Suicin 3908	
		Present or absent	Number of contigs	Present or absent	Number of contigs	Present or absent	Number of contigs
NSUI001	25	Mostly present^a^	1	Absent	N/A	Absent	N/A
NSUI006	25	Mostly present	1	Absent	N/A	Absent	N/A
NSUI012	25	Mostly present	1	Absent	N/A	Absent	N/A
NSUI033	25	Present^b^	1	Absent	N/A	Absent	N/A
NSUI034	25	Present	1	Absent	N/A	Absent	N/A
NSUI035	25	Present	2	Absent	N/A	Absent	N/A
NSUI037	25	Present	1	Absent	N/A	Absent	N/A
NSUI038	25	Present	1	Absent	N/A	Absent	N/A
NSUI039	25	Present	1	Absent	N/A	Absent	N/A
NSUI040	25	Present	1	Absent	N/A	Absent	N/A
NSUI041	25	Mostly present	2	Absent	N/A	Absent	N/A
NSUI042	25	Mostly present	1	Absent	N/A	Absent	N/A
NSUI043	25	Mostly present	1	Absent	N/A	Absent	N/A
NSUI044	25	Mostly present	2	Absent	N/A	Absent	N/A
NSUI045	25	Mostly present	2	Absent	N/A	Absent	N/A
NSUI046	25	Mostly present	2	Absent	N/A	Absent	N/A
NSUI047	25	Mostly present	1	Absent	N/A	Absent	N/A
NSUI048	25	Present	1	Absent	N/A	Absent	N/A
NSUI049	25	Mostly present	1	Absent	N/A	Absent	N/A
NSUI050	25	Absent^c^	N/A^e^	Absent	N/A	Absent	N/A
NSUI051	25	Absent	N/A	Absent	N/A	Absent	N/A
NSUI052	25	Mostly present	1	Absent	N/A	Absent	N/A
NSUI053	25	Mostly present	1	Absent	N/A	Absent	N/A
NSUI054	25	Mostly present	1	Absent	N/A	Absent	N/A
NSUI055	25	Mostly present	1	Absent	N/A	Absent	N/A
NSUI056	25	Mostly present	1	Absent	N/A	Absent	N/A
NSUI057	25	Mostly present	1	Absent	N/A	Absent	N/A
NSUI060	25	Mostly present	1	Absent	N/A	Absent	N/A
NSUI061	25	Mostly present	1	Absent	N/A	Absent	N/A
NSUI063	25	Mostly present	1	Absent	N/A	Absent	N/A
NSUI065	25	Mostly present	1	Absent	N/A	Absent	N/A
NSUI066	25	Mostly present	1	Absent	N/A	Absent	N/A
NSUI068	25	Mostly present	2	Absent	N/A	Absent	N/A
NSUI069	25	Present	1	Absent	N/A	Absent	N/A
NSUI070	25	Present	1	Absent	N/A	Absent	N/A
NSUI072	25	Mostly present	1	Absent	N/A	Absent	N/A
NSUI075	25	Mostly present	3	Absent	N/A	Absent	N/A
NSUI077	25	Present	1	Absent	N/A	Absent	N/A
NSUI078	25	Mostly present	1	Absent	N/A	Absent	N/A
NSUI082	25	Absent	N/A	Absent	N/A	Absent	N/A
NSUI088	25	Present	1	Absent	N/A	Absent	N/A
NSUI089	25	Mostly present	1	Absent	N/A	Absent	N/A
NSUI092	25	Present	1	Absent	N/A	Absent	N/A
NSUI093	25	Mostly present	1	Absent	N/A	Absent	N/A
NSUI094	25	Mostly present	1	Absent	N/A	Absent	N/A
NSUI096	25	Mostly present	1	Absent	N/A	Absent	N/A
NSUI097	25	Present	2	Absent	N/A	Absent	N/A
NSUI099	25	Present	1	Absent	N/A	Absent	N/A
NSUI100	25	Mostly present	3	Absent	N/A	Absent	N/A
NSUI102	25	Mostly present	1	Absent	N/A	Absent	N/A
NSUI103	25	Mostly present	1	Absent	N/A	Absent	N/A
NSUI002	28	Absent	N/A	Absent	N/A	Absent	N/A
NSUI003	28	Absent	N/A	Present	1	Absent	N/A
NSUI004	28	Absent	N/A	Absent	N/A	Absent	N/A
NSUI005	28	Absent	N/A	Present	1	Absent	N/A
NSUI007	28	Absent	N/A	Present	2	Absent	N/A
NSUI008	28	Absent	N/A	Absent	N/A	Absent	N/A
NSUI009	28	Absent	N/A	Present	1	Absent	N/A
NSUI010	28	Absent	N/A	Absent	N/A	Absent	N/A
NSUI011	28	Absent	N/A	Absent	N/A	Absent	N/A
NSUI013	28	Some genes^d^	N/A	Absent	N/A	Absent	N/A
NSUI014	28	Absent	N/A	Absent	N/A	Absent	N/A
NSUI015	28	Absent	N/A	Present	1	Absent	N/A
NSUI016	28	Absent	N/A	Present	1	Absent	N/A
NSUI017	28	Absent	N/A	Present	1	Absent	N/A
NSUI018	28	Absent	N/A	Mostly present	1	Absent	N/A
NSUI019	28	Absent	N/A	Absent	N/A	Absent	N/A
NSUI020	28	Absent	N/A	Absent	N/A	Absent	N/A
NSUI021	28	Absent	N/A	Absent	N/A	Absent	N/A
NSUI022	28	Absent	N/A	Mostly present	2	Absent	N/A
NSUI023	28	Absent	N/A	Mostly present	2	Absent	N/A
NSUI024	28	Absent	N/A	Mostly present	2	Absent	N/A
NSUI025	28	Absent	N/A	Present	2	Absent	N/A
NSUI026	28	Absent	N/A	Mostly present	1	Absent	N/A
NSUI027	28	Present	1	Absent	N/A	Absent	N/A
NSUI028	28	Present	1	Absent	N/A	Absent	N/A
NSUI029	28	Absent	N/A	Absent	N/A	Absent	N/A
NSUI030	28	Present	1	Absent	N/A	Absent	N/A
NSUI031	28	Absent	N/A	Absent	N/A	Absent	N/A
NSUI032	28	Absent	N/A	Absent	N/A	Absent	N/A
NSUI036	28	Absent	N/A	Mostly present	1	Mostly present	1
NSUI058	28	Absent	N/A	Absent	N/A	Absent	N/A
NSUI059	28	Present	1	Absent	N/A	Absent	N/A
NSUI062	28	Absent	N/A	Absent	N/A	Absent	N/A
NSUI064	28	Absent	N/A	Absent	N/A	Absent	N/A
NSUI067	28	Present	1	Absent	N/A	Absent	N/A
NSUI073	28	Absent	N/A	Absent	N/A	Absent	N/A
NSUI074	28	Absent	N/A	Absent	N/A	Absent	N/A
NSUI076	28	Absent	N/A	Absent	N/A	Absent	N/A
NSUI079	28	Absent	N/A	Absent	N/A	Absent	N/A
NSUI080	28	Absent	N/A	Present	3	Absent	N/A
NSUI081	28	Absent	N/A	Absent	N/A	Absent	N/A
NSUI083	28	Absent	N/A	Absent	N/A	Absent	N/A
NSUI084	28	Absent	N/A	Absent	N/A	Absent	N/A
NSUI085	28	Present	1	Absent	N/A	Absent	N/A
NSUI086	28	Present	1	Absent	N/A	Absent	N/A
NSUI087	28	Absent	N/A	Absent	N/A	Absent	N/A
NSUI090	28	Absent	N/A	Absent	N/A	Absent	N/A
NSUI091	28	Absent	N/A	Present	1	Absent	N/A
NSUI095	28	Present	2	Absent	N/A	Absent	N/A
NSUI098	28	Absent	N/A	Absent	N/A	Absent	N/A
NSUI101	28	Absent	N/A	Absent	N/A	Absent	N/A

^a^The majority of the genes are present, but some are missing or truncated.

^b^The suicin gene cluster is present in its entirety.

^c^The suicin gene cluster is completely absent.

^d^The majority of the genes are absent, but some are still present.

^e^N/A: not applicable.
